# The Use and Abuse of the Brain

**Published:** 1896-03

**Authors:** 


### The Use and Abuse of the Brain.

In the course of an address on this subject, Dr. Wm. A. Hammond recently said: "Anxiety causes more brain disorders than any other agency I know of, unless it be love. Many jokes are made about the gray matter of the brain, but I will say right here that I have a great respect for the gray matter of the brain. There is no higher organism than that. It is the grandest organ in man, and were I ever to worship anything, it would be a portion of the gray matter of the brain. It is well for us to know that the emotions cause more unhappiness and crime than any other function of the brain. Human beings are governed by their emotions, and it is well that they should be, though it is the emotions that wear away the brain, and not honest, intellectual work. Very few people suffer from intellectual work, and if my memory serves me I do not recollect ever having a mathematician for a patient. It is not intellectual work that causes nervous dyspepsia, but the emotions, such as anxiety, fear, sorrow, and love. I consider that eight hours are sufficient for a man to use his brain, because if he exceeds that time he becomes nervous and fretful, and an exhaustive brain is an irritable brain. You may not feel the evil effects of the stress of brain work at the time, but you will sooner or later, when it is too late. The men that work at night with their brains are the ones that expose themselves to danger and death, which will surely come unless the great strain on the mind is lightened."

